# COMPARISON OF THE ACTIVITIES OF FOUR ANTIFUNGAL AGENTS IN AN *IN VITRO* MODEL OF DERMATOPHYTE NAIL INFECTION

**DOI:** 10.4103/0019-5154.43216

**Published:** 2008

**Authors:** Hossein Nowrozi, Golrokh Nazeri, Parvaneh Adimi, Mohsen Bashashati, Masood Emami

**Affiliations:** *From the Iran University of Medical Sciences, Tehran, Iran*; 1 *From the Gom Azad University, Gom, Iran*; 2 *From the Tehran University of Medical Sciences, Tehran, Iran*; 3 *From the Karaj Azad University, Karaj, Iran*

**Keywords:** *Antifungal*, *culture media*, *nail*, *onychomycosis*

## Abstract

**Background:**

Onychomycosis is a difficult condition to treat and cure rates are disappointing. Moreover fungicidal action of antifungal agents in NCCLS assays and their rapid accumulation in nails *in vivo* are not compatible with the duration of treatment.

**Aims:**

This study aimed to find the effectiveness of 4 different antifungal agents in an *in vitro* model with some similarities to *in vivo* conditions.

**Materials and Methods:**

Strains of Trichophyton rubrum I-III, Trichophyton mentagrophytes (usual form), Trichophyton mentagrophytes 73, Epidermophyton Flucosom, Microsporum Canis, and Trichophyton Schoenleini which were isolated from the nails of patients, were hired. Inocula suspensions were prepared from 7 to 14 day-old cultures of dermatophytes. Antifungal agents including fluconazole, ketoconazole, terbinafine, and griseofulvin were obtained as standard powders. For each antifungal agent, initial MIC was calculated by registering the optical density for 10 two-fold serially diluted forms which was incubated with diluted fungal suspensions with RPMI 1640. Human nail powder inoculated with different strains and incubated in RPMI 1640 and different concentrations of antifungal drugs for 4 weeks. Final MIC at different steps of 1, 2, 3 and 4 weeks were investigated.

**Results:**

The final MIC that resulted from the incubation of dermatophytes with nail powder was much more than the initial which was concluded from conventional MIC assay. Terbinafine had the lowest rate of initial and final MICs.

**Conclusion:**

The model described here may present more similar conditions to clinical fungal infections; therefore the results such as MIC may be more helpful for hiring the most effective antifungal agent.

## Introduction

Onychomycosis (OM) is fungal infection of the toenails or the fingernails. It is not life-threatening, however it can cause pain, discomfort, and deformity and may have a significant effect on the quality of life.^1^ Half of all nail disorders are due to OM. Moreover, 30 percent of patients with a cutaneous fungal infection also have OM. Additionally, the incidence of OM has been increasing, on account of increasing in the incidence of diabetes mellitus, immunosuppression and increasing age.^2^

Despite fungicidal effect of anti-fungal drugs (e.g. terbinafine) in National Committee for Clinical Laboratory Standards (NCCLS) assays and their rapid accumulation in nails *in vivo*, onychomycosis patients require prolonged treatment to be cured.^3^ Therefore, an *in vitro* model with some similarities with *in vivo* conditions might help us to assess this dilemma. In addition, since this infection is very common, especially in elderly patients, effective topical therapy would provide an outstanding treatment for such patients.^4^

We decided to investigate on the antifungal drug response of 4 different agents in an *in vitro* model of dermatophyte nail infection. To investigate this, as introduced by Osborne *et al.*^3^ and Yazdanparast *et al*,^5^ we used a more clinically relevant onychomycosis *in vitro* test. To provide this condition, human nail powder inoculated with growth media and provided an extensive and invasive fungal growth media.

## Materials and Methods

Standard methods were used for preparation of the stock organisms, stock solution of antifungal agents, and MIC determination.^6^–^9^

**Organism:** A total of 3 strains of Trichophyton (T) rubrum (I,II,III), 2 strains of Trichophyton mentagrophytes (usual form and, T. mentagrophytes 73), Epidermophyton (E) Flucosom, Microsporum (M) Canis, M. Gypseum and T. Schoenleini which were isolated from the nails of patients, were hired from the department of mycology and Health Research Institute in Tehran University of Medical Sciences.

**Stock Inocula preparation:** Inocula suspensions were prepared from 7 to 14-day-old cultures of dermatophytes grown on Potato Dextrose Agar at 30°C. Briefly, colonies were covered with 2 ml of sterile saline, and then rubbed carefully with the tip of a Pasteur pipette. Heavy particles of the suspension were allowed to settle for 3 to 5 min. The final inoculum size was adjusted with a spectrophotometer at a wavelength of 530 nm to a transmittance of 95%. These suspensions were diluted in RPMI 1640 test medium (1:50) to obtain a cell number ranging from 0.5 × 10^4^ to 5 × 10^4^ CFU/ml.

**Antifungal agents:** They were obtained as standard powders. The powder of fluconazole 99.7% (Pars Daru, Iran), ketoconazole 98.43% (Rooz Daru, Iran), terbinafine 99.6% (Behvarzan, Iran), and griseofulvin 99.3% (Daroopakhsh, Iran) were used in this study.

Stock solutions were prepared by dissolving and two-fold serially dilution of drugs in Methanol 0.1 percent, by using Broth microdilution method. 10× dilutions were prepared for each drug Tested concentrations for fluconazole ranged from 0.187-96.0 µg/ml, and for ketoconazole, terbinafine and griseofulvin from 0.125-64.0 µg/ml. Stock solutions were prepared by dissolving and two-fold serially dilution of drugs in Methanol 0.1 percent.

**Determination of initial MIC:** For each fungal strain 13 tubes with the volume of 10 ml, were prepared. 0.1 cc of each antifungal agent dilutions were added to tubes 1 to 10. Then 0.9 cc of diluted fungal suspensions with RPMI 1640 was added to each tube. The eleventh tube contained a 0.9 ml volume of inoculum suspension and a 0.1 ml volume of drug-free medium (To assess the inhibitory effect of medium on fungal growth). The twelfth tube (growth control) consisted of fungal suspensions with RPMI 1640. A sterility control (the thirteenth tube) was run in parallel by including a 1 ml volume of uninoculated, drug-free medium.

Tubes were incubated at 30°C for 48h. Growth control tubes were observed for the presence or absence of visible growth. When growth was visible, the growth in each tube was compared with that of the growth control tube. Optical density (OD) of each tube which was obtained from a spectrophotometer at a wavelength of 530 nm was used to find the amount of reduction in turbidity as compared to that of the drug-free control tube. MIC at which 50% of the isolates are inhibited (MIC_50_) was determined.

**Preparation of nail powder:**^5^,^10^ Human nail Clippings from several healthy volunteers previously found free from fungi when examined in 10% KOH were used. The samples were cleaned with 70% ethanol, dried in a sterile Petri dish at 37°C, and ground with using a stainless steel peppermill. The powder was autoclaved at 121°C for 20 min.

**Nail model culture:** After finding the primary MIC for each microorganism, 6 tubes containing MIC tube, 2 tubes with higher dilution and 2 tubes with lower dilution, respectively plus another tube as the control tube were selected. Autoclaved nail powder was added, at approximately 10 mg per tube. 0.9 cc of Inocula suspensions were added directly onto the nail powder. The tubes were left at 37°C for 10 days. After 10 days the mycelium were attached to nail particles which established the sufficiency of the growth time. 0.1 cc of antifungal agents were added to the culture according to the calculated MIC, and the cultures were returned to 37°C. Final MIC 4 weeks after incubation with nail powder was investigated.

### Statistical analysis

Initial and final MICs were assessed 2 times for each sample and presented as means. ANOVA besides Tukey test was hired to analyze MICs in each subgroup. Pearson correlation was used in order to find any correlation between initial and final MICs. Paired t-test was used for comparing between initial and final MICs.

## Results

Initial MICs (before culturing on nail powder) for each subgroup are presented in Table 1.

**Table 1:** Initial MIC* for different antifungal agents according to the type of dermatophytes

	Ketoconazole	Fluconazole	Terbinafine	Grisofulvin
Dermatophytes				
*T. rubrum I*	32	48	2	32
*T. rubrum II*	16	24	4	16
*T. rubrum III*	32	48	2	32
*T. mentagrophytes*	16	48	0.125	8
*T. mentagrophytes73*	32	48	1	32
*T. schoenleini*	0.5	48	4	0.5
*E. flucosom*	1	48	2	16
*M. canis*	8	24	8	16
*M. gypseum*	16	24	8	64
MIC range	0.5-32	24-48	0.125-8	0.5-32

* µg/ml

Grisofulvin and ketoconazole had the same rate of initial MICs, which were statistically different from fluconazole with the highest rate of initial MIC and terbinafine with the lowest rate of initial MIC respectively (ANOVA, *P* = 0.0001).

After incubation with nail powder we found that final MICs statistically increased in comparison with initial MICs (Initial MIC: 19.42 ± 17.21 µg/ml, final MIC 36.72 ± 33.77 µg/ml, paired *t* -test, *P* value = 0.0001) (Table 2).

**Table 2:** Final MIC for different antifungal agents according to the type of dermatophytes

	Ketoconazole	Fluconazole	Terbinafine	Grisofulvin
Dermatophytes				
*T. rubrum I*	64	96	4	32
*T. rubrum II*	16	48	8	32
*T. rubrum III*	64	96	4	32
*T. mentagrophytes*	64	96	1	16
*T. mentagrophytes73*	-	96	4	32
*T. schoenleini*	2	96	16	1
*E. flucosom*	2	96	2	32
*M. canis*	16	48	16	16
*M. gypseum*	32	-	32	-

There was a direct correlation between initial and final MICs which was statistically significant (R = 0.95, *P* value = 0.0001).

## Discussion

Griseofulvin and newer antifungal drugs such as ketoconazole, fluconazole and terbinafine, are the major systemic drugs applied to treat onychomycosis.^11^ Griseofulvin was the first systemic antifungal drug but nowadays it is not used in a widespread way.^12^ Ketoconazole was the first orally active imidazole but it is known for its hepatotoxicity as well.^7^ Terbinafine is highly effective against dermatophyte infections and acts by blocking ergosterol synthesis.^10^

Studies which compared the effectiveness of different antifungal agents in treatment of onychomycosis at the same time^7^ are scarce. Moreover, treatment of OM is a problematic issue in clinical practice.^1^,^10^

Santos *et al*,^7^ tried to study *in vitro* susceptibility of 52 isolates of Trichophyton rubrum and 40 of Trichophyton mentagrophytes to griseofulvin, terbinafine, itraconazole, ketoconazole, fluconazole and cyclopiroxolamine. They used modified NCCLS approved procedure M38-A. Finally, they concluded that terbinafine was the most effective *in vitro* against all isolates, followed by itraconazole, cyclopiroxolamine, ketoconazole and fluconazole.

Conidial suspensions or mixed suspension of mycelial fragments and conidia growing in a rich medium are used in the NCCLS methodology for antifungal testing makes use of.^13^,^14^ Employing nail powder for antifungal testing,^10^ mimics the course of a natural nail fungal infection, with antifungal treatment starting only after mycelial growth. In this method the fungi must use the nails as their only nutrition by activation of keratinases secretion. In this *in vitro* model, using its natural substrate, the dermatophytes act with more similarity to *in vivo* clinical condition.^5^,^10^,^15^,^16^ Therefore, response to antifungal agents which is normally reported as MIC reached form NCCLS models would reach more relevant and practical result.

Due to our findings, the final MIC which resulted after the incubation of dermatophytes beside nail powder was much more than the initial which was concluded from conventional MIC assay. In addition Osborne *et al*,^10^ found that terbinafine Nail-MFCs obtained from *in vitro* method using nail powder were much higher than MFC values obtained after conventional assays. Also, they found that the cidal action of terbinafine was much slower than in conventional assays. Therefore the current model of culture may show the needs for high dose and long duration of antifungal treatment for OM.

On the other hand, by comparing the efficacy of the assessed antifungal drugs, we found that terbinafine inhibited the growth of dermatophytes in the *in vitro* model more effectively. Osborne *et al*, concluded similarly.^10^ The higher activity of terbinafine in comparison to other tested antifungal drugs has been established during *in vitro* studies.^7^,^17^ Therefore our finding in this regard is not new rather than higher MIC which had been also achieved by Osborne *et al.*^10^

In conclusion, the model described here offers a clinically mimicking condition for measuring the efficacy of different antifungal drugs for the treatment of onychomycosis. Future studies on dermatophytes using other biomaterials such as hair powder as a part of culture medium may be useful for other clinical conditions.
